# Population structures of the water-borne plant pathogen *Phytopythium helicoides* reveal its possible origins and transmission modes in Japan

**DOI:** 10.1371/journal.pone.0209667

**Published:** 2018-12-26

**Authors:** Auliana Afandi, Emi Murayama, Yin-Ling, Ayaka Hieno, Haruhisa Suga, Koji Kageyama

**Affiliations:** 1 The United Graduate School of Agricultural Science, Gifu University, Gifu, Japan; 2 Biotechnology Study Program, Graduate School of Universitas Gadjah Mada, Yogyakarta, Indonesia; 3 River Basin Research Center, Gifu University, Gifu, Japan; 4 Inner Mongolia University for Nationalities, Tongliao, China; 5 Life Science Research Center, Gifu University, Gifu, Japan; University of Innsbruck, AUSTRIA

## Abstract

The purpose of this study was to clarify the genetic diversity of *Phytopythium helicoides* and to understand the transmission mode of the pathogen in Japan. In total, 232 *P*. *helicoides* isolates were collected from various host plants and geographic origins, including farms and natural environments. We developed 6 novel microsatellite markers for use in the study and found 90 alleles among the 6 markers in the 232 isolates. The analysis of molecular variance suggested that *P*. *helicoides* has high variance within individuals and low fixation indices between populations. A phylogenetic analysis revealed that isolates collected from the same hosts and/or geographic origins were often grouped together. For example, several isolates from natural environments were grouped with isolates from nearby agricultural areas. On the other hand, 2 geographically distant populations collected from the same host plant had similar genotypes. Our results suggested that migration of the pathogen could be facilitated naturally via drainage systems or by human activity in the transport of agricultural materials.

## Introduction

Developments in agriculture and global trading, and innovations in plant breeding can lead to the wide distribution of genetically homogeneous crops. These factors, along with the tendency of farmers to cultivate the same varieties over large areas could accelerate the pace of pathogen dispersal to new areas. Both human mediated introduction and natural dispersal mechanisms can lead to the spread of pathogens [1]. Humans can accidentally introduce pathogens to new areas through the transport of infected agricultural materials. In Japan, the widespread use of hydroponic culture systems could lead to the rapid spread of water borne diseases. *Phytopythium helicoides* is a soil and water-borne pathogen that causes serious problems in a variety of horticultural crops. This pathogen produces large numbers of zoospores that serve as a secondary inoculum source. Thus, *P*. *helicoides* can spread rapidly in hydroponic farming systems.

*Phytopythium* is a recently established genus consisting of species that were formerly classified in the *Pythium* phylogenetic clade K [2]. These species are morphologically similar to *Pythium* spp. but are genetically closer to *Phytophthora* spp. [3]. *Phytopythium helicoides* is representative species that produces papillate sporangia like *Phytophthora spp*. It also has a *Pythium*-like zoospore discharge mechanism whereby the plasma flows out of the sporangium through a discharge tube to form a plasma-filled vesicle at the tip. *Phytopythium helicoides* is a high temperature tolerant pathogen that was first isolated from dahlia roots in 1930 by Drechsler in the USA[4]. After that, there were few reports of the pathogen until it was isolated on miniature rose in Gifu, Japan [4]. Since then it has caused root and stem rot in other agricultural important plants in Japan, including kalanchoe, kiwi, and strawberry [5,6]. Furthermore, it has recently caused root rot and stem rot in bell pepper and pistachio in the USA [7], citrus mandarin and kiwi fruit in china [8,9], and rose in Korea [10]. These diseases suddenly appeared in areas where they had not occurred before, however, the sources of the inoculum have not been identified.

As clonal reproduction and selfing is common in oomycetes [11], we would expect clonal population in the relatively close sampling area or similar host origin. *Phytopythium helicoides* produces asexual structures such as zoospores to swim in water and to infect another host plant. Furthermore, if the infected plants have died, it produces sexual structures such as oospores to make recombination and to survive in soil for a long time. Previous studies on the genetic diversity of *P*. *helicoides* revealed high variability in the rDNA ITS region within a single isolate, suggesting that this pathogen will undergo cross-breeding even though it is a homothallic species [12]. Despite its ability to cross-breed, this pathogen can also lose its ability to produce sexual structures. Asexual strains of *P*. *helicoides* produce no oospores but abundant sporangia. The sporangium is an effective survival structure in the greenhouse, where high humidity and temperatures are maintained throughout the year [13]. The high variability among isolates of *P*. *helicoides* could indicate that new strains will emerge in the future.

Understanding the genetic variability in a pathogen could help in the development of effective disease management strategies. Studies that address the microevolution and population structure of a pathogen are necessary to predict its adaptation and migration abilities. A pathogen that has high genetic diversity and high mobility is likely to adapt to environmental change. There are many methods for studying population genetics at the molecular level, and they are generally based on DNA polymorphisms such as single nucleotide polymorphisms or microsatellites. Microsatellites (also called simple sequence repeats) are very convenient for molecular studies involving PCR because they are codominant, multiallelic, and highly polymorphic, and only small amounts of DNA are needed for PCR analysis [13]. We used microsatellites as a primary source of genotyping data in this study. Our aims were to (i) develop reliable microsatellite markers for *P*. *heliocoides*; (ii) identify the main genotypic clusters of *P*. *helicoides* in Japan; (iii) calculate the genetic diversity within the *P*. *helicoides* populations, and (iv) understand the transmission modes of *P*. *helicoides* across Japan.

## Materials and methods

### Isolates and DNA extraction

**Fig 1:**
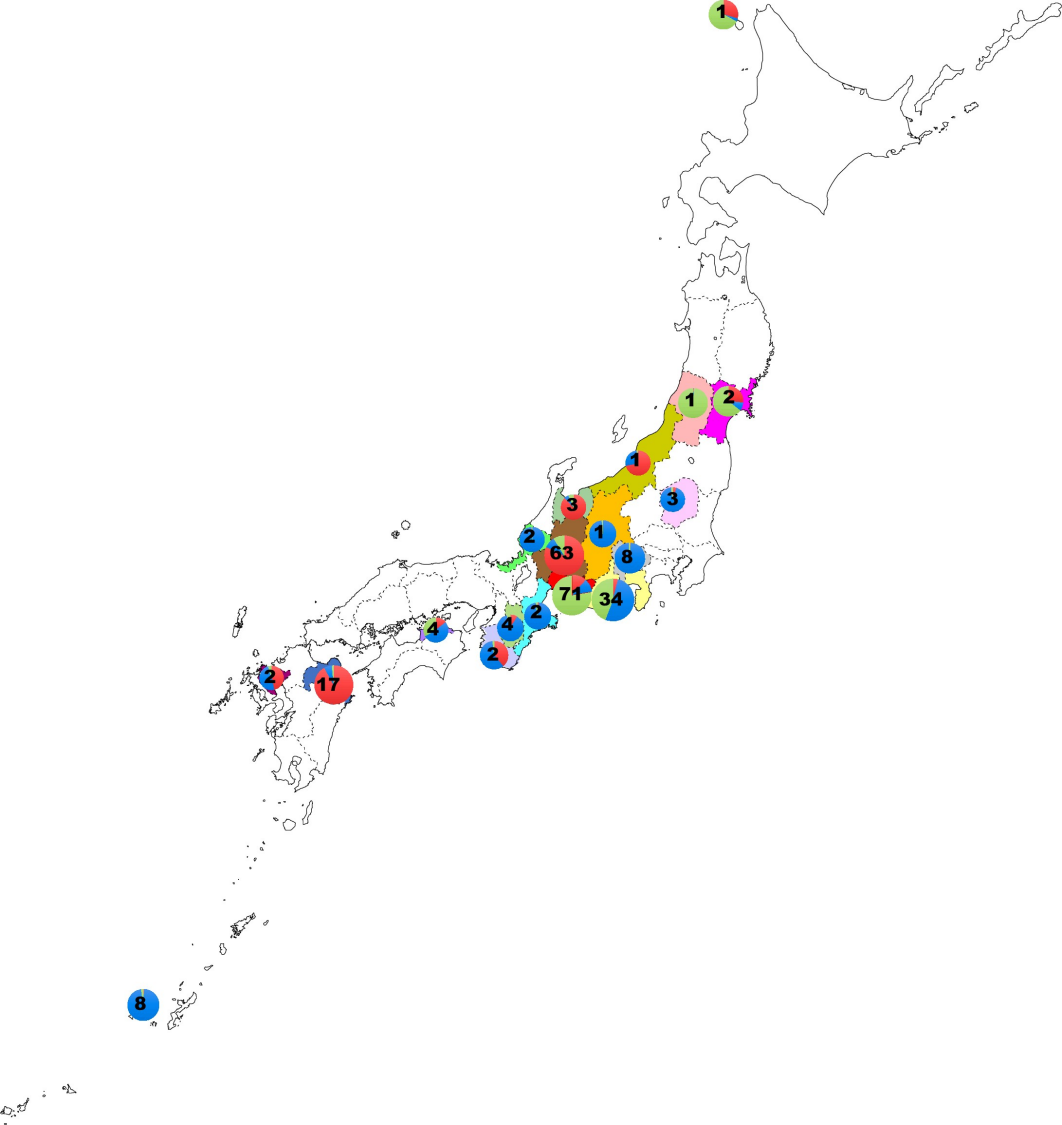
Prefectures in Japan where *P*. *helicoides* isolates were collected, with numbers of isolates collected. Pie diagrams indicate the genetic clustering of each population based on the STRUCTURE analysis. ■ Aichi; ■ Fukui; ■ Gifu; □ Hokkaido ■ Iriomote; ■Kagawa; ■ Mie; ■ Miyagi; ■ Nagano; ■ Nara; ■ Niigata ■Oita; ■ Saga; ■Shizuoka; ■ Tochigi; ■ Toyama; ■ Wakayama ■ Yamagata ■ Yamanashi.

No specific permissions were required for this study, and no endangered or protected species were involved. In total, 232 isolates of *P*. *helicoides* were selected from the Gifu University Culture Collection and used in this study (S1 Table). Of these, 229 were collected from 19 geographical regions in 19 prefectures of Japan (Fig 1), 2 were from the USA, and one came from the Netherlands. Most of the isolates were collected from the plants, soil, or water used in the production of horticultural and ornamental crops. However, 20 isolates were collected from natural environments outside agricultural areas. To understand the population structures within local areas, we studied 49 isolates from several poinsettia farms in Aichi, 30 isolates from six miniature rose farms in Gifu, and 22 isolates from three cutting rose farms in Shizuoka. All isolates were stored on corn meal agar medium at room temperature until DNA extraction.

DNA was extracted using the PrepMan Ultra Sample Preparation Reagent (Applied Biosystems). First, each isolate was cultured on V8 medium [14] to increase the number of mycelia produced. The mycelium was harvested by directly transferring it to 200 ml reagent-water suspension (1:1) and DNA was extracted using the manufacturer’s protocol.

### Microsatellite marker development and PCR reactions

The microsatellite markers were developed using the suppression PCR and thermal asymmetric interlaced (TAIL)-PCR methods, as described by Yin-Ling et al. (2009) [15]. Briefly, the suppression step was performed as follows: Genomic DNA of isolate GUCC 5015 was digested with AluI (Toyobo), then the fragments were purified and ligated to two adaptors: a 48-mer (5’-GTAATACGACTCACTATAGGGCACGCGTGGTCGACGGCCCGGGCTGGT-3’) and an 8-mer with the 3’ end capped by an amino residue (5’-ACCAGCCC-NH_2_-3’). The resulting DNA library was then used as the template in PCR amplifications using the adapter primer AP2 (5’-CTATAGGGCACGCGTGGT-3’) and one of 6 microsatellite primers: (AGC)_10_, (CAA)_10_, (CTT)_10_, (GGA)_10_, (TCA)_10_, and (TGC)_10_. The PCR products were cloned using the TOPO TA cloning kit (Invitrogen). After culturing, individual colonies were picked and purified using ExoSAP-IT kit (Affimetrix). The purified PCR product was sequenced using M13M4 primer and the BigDye Terminator Sequencing Kit (Thermo Fisher Scientific) on an ABI3500 automated sequencer (Applied Biosystem). TAIL-PCR was then used to develop primers which would amplify the flanking regions of the microsatellite markers. Three sense primers (a, b, and c) were designed for each sequence obtained in the suppression step (S2 Table). The sense primers were used in 3 consecutive PCR reactions (c, b, then a) with the arbitrary degenerate primer AD4 (5'-gtNcgaSWcaNaWgtt-3')[14] that binds to various places in the genome. The first PCR was performed in 25 μl with 20 ng genomic GUCC 5015 DNA, 0.2 μM primer c, 5 μM AD4 primer, 0.5 Units TaKaRa Taq DNA polymerase (Takara Bio Inc.), 0.2 mM dNTPs, and 1 × PCR buffer (10 mM Tris-HCl (pH 8.3), 50 mM KCl, 1.5 mM MgCl_2_). The second PCR was performed in 25 μl with 1 μl of a 50-fold dilution of the products of the first reaction, 0.2 μM primer b, 3 μM AD4 primer, 0.5 Units TaKaRa Taq DNA polymerase, 0.2 mM dNTPs, and 1 × PCR buffer. The third PCR was performed in 50 μl with 1 μl of a 50-fold dilution of the products of the second reaction, 0.2 μM primer a, 0.2 μM AD4 primer, 3.5 Units TaKaRa Taq DNA polymerase (Takara Bio Inc.), 0.2 mM dNTPs, and 1 × PCR buffer.

Major bands from the TAIL-PCR reactions were sequenced as described above and used to design specific PCR primers that flanked the microsatellites using Primer-BLAST (https://www.ncbi.nlm.nih.gov/tools/primer-blast/). These and 5 primer sets from a previous study [15]were tested with 3 strains of *P*. *helicoides* (GUCC 5056, GUCC 5076, and GUCC 5135). PCR reactions were carried out, and the products were cloned and sequenced using standard procedures.

Six primer pairs were selected for PCR analysis of all 232 *P*. *helicoides* isolates. Reactions were performed in a total volume of 25 μl containing 2 μL of 1 ng/μL DNA, 2.5 μL of 10 × PCR Buffer (plus Magnesium, Takara), 2.5 μl of 4 mg/mL BSA, 2.5 μL each of 10 mM forward and reverse primers, 2 μL of 2.5 mM dNTP mix (Takara), 0.1 μL rTaq polymerase (Takara), and 10.9 μL H_2_O. The following conditions were used: 94°C for 5 min; 35 cycles of 94°C for 30 s, 50 or 60 ^o^C for 30 s, and 72 ^o^C for 30 s; then 72 ^o^C for 7 min. The products were assessed by GelRed staining (Biotium) using 2% agarose gels in 0.5 × Tris-Acetate-EDTA buffer, and bands were visualized under UV light. The fragments were analyzed on an ABI3100 or ABI3130 Genetic Analyzer (Applied Biosystem) using the LIZ 250 DNA ladder as a marker. The electropherograms were scored manually.

### Locus characteristics and diversity analysis

The numbers of alleles, numbers of unique alleles, and observed and expected heterozygosities were calculated using GenAlex v 6.503 [16,17]. The analysis of molecular variance as well as the fixation index was investigated using the same program with 9999 permutations. Populations that consisted of only one sample were eliminated to make the calculation possible. Each locus was tested for the null alleles using Microchecker [18]. The Genepop package in R [19] was used to test each locus for its Hardy Weinberg Equilibrium (HWE) status and its pairwise linkage disequilibrium status. All *P*-values were corrected using the Holm–Bonferroni method.

Basic population statistic were also calculated on the R package poppr [20], including number of multi locus genotype (MLG), number of expected MLG (e-MLG), the Hardy Weinberg Equilibrium, as well as Index of association [21].

### Population structure analysis

Population structures were analyzed using a cluster analysis, performed by estimating the probabilities of genotypes being distributed into K clusters using STRUCTURE v. 2.3.4. [22–25]. Nine independent runs were performed for each K = 1–20. Each run assumed population admixture for correlated allele frequencies with 200000 burn-in lengths followed by 100000 repetitions of Markov chain Monte Carlo (MCMC). The optimal number for K was determined using STRUCTURE HARVESTER[26] and matched up from the independent runs using CLUMPP[27]. The result was finally visualized using DISTRUCT [28].

A distance based phylogenetic tree was constructed using Neighbour- Joining algorithm with 1000 boothstrap iterations on the R environment using package ape[29]. The tree data were then exported in newick format and processed using fig tree (http://tree.bio.ed.ac.uk/software/figtree/). The tips of the tree were labelled with different color and shape based on its geographic and host origin, respectively, for easier interpretation.

## Results

### Development of microsatellite markers and diversity analysis of *P*. *helicoides*

We obtained amplified products from 27 of the primer sets. After sequencing we found that 7 primer sets amplified microsatellite regions, and that two additional motifs, (AGGCA)n and (GCAGAC)n, were observed among the regions. We used those 7 amplicons to design specific PCR primers that flanked the microsatellites. These primers along with 5 primer pairs from a previous study [15] were used in PCR reactions with 3 strains of *P*. *helicoides* (GUCC 5056, GUCC 5076, and GUCC 5135) as templates (Table 1). The PCR products were cloned and sequenced. Some primers amplified more than 2 alleles from a single isolate and would not be useful in population genetics studies. Two primer pairs amplified monomorphic regions from all three isolates, and these were also discarded. We identified 6 primer pairs that were suitable for population genetics analyses. The sequences of these primer pairs are shown in Table 1.

**Table 1:** Selection of microsatellite markers used in this study.

No	Primer	Primer sequence(5`-3`)	Amplicon size		
			GUCC5056	GUCC5076	GUCC5135
1	YL-AG^a^	F: CCAGCATCCACGGCAATC	117, 119, 121	117, 119, 121, 123	117, 119, 121
		R: GCACATATTCCATTCGACCTG			
2	YL-AGC^a^	F: CCAGGATTGAGCTAGTAGCAGT	285	285	285
		R: ACCGAAGTTACGAAGACG			
3	YL-CAA^a^	F:GAACCCAAGCAGTTTCCTGTTAGC	519	519	519
		R: GACCGCAACGCCCTCAAAACG			
4	YL-CTTT^a^	F: ACACCAACCATATGCTTT	151, 171, 175, 179, 183, 187, 191, 195, 199, 203, 215	151, 155, 163, 167, 171, 175, 179, 183, 187, 191, 195, 199, 203, 207, 215	147, 163, 167, 171, 175, 179, 183, 187, 191, 199
		R: GTCATCCTCGTACTTTCT			
5	YL-TCA^a^	F: GCAATCACAGCTCCCACA	166, 184, 187	175, 190	187
		R: AGAAGTAGCGTTGGAAAGA			
6	EM-AGC1	F: CCGAGTCTACACCAACATGTTCACC	71, 77	71, 80	71,77
		R: TGCGTCTGCATCTGTGCGTG			
7	EM-CTT2	F: TCGAAGAATCTCGCCAACCACC	126, 141	117, 138, 144	111, 114, 117, 123, 141, 144
		R: GCGACAACATGGATGCTCGTG			
8	EM-GGA1	F: AGCAGGGTTTGTTGCTGGAAG	77, 80	86	77, 86
		R: ACGATCCCTCCGCCATATCC			
9	EM-GGA2	F: GTGACGAGAATTCGAGCGTGTG	126, 138	138	68
		R: TGGTGGATGGATCTCTTCAACCTAC			
10	EM-AGGCA1	F: CGAATGGATATCGGCACGCC	78,88	94	100
		R: TGGGTCTGCCAATGGGTCTG			
11	EM-CTT1	F: GCATTTCCAAGAGGAACCCGCC	55, 70	55, 70	70
		R: ATGGGGCAAGTCCAGCCCAAAAG			
12	EM-GCAGAC	F: ACCTCGGTGACAGCAGTGATC	94	94	76,94
		R: AGGCTTCTGCGGTGTCTACG			

^a^: Yin-Ling, et al., 2009

We analyzed all 232 isolates of *P*. *helicoides* using the selected primer pairs, which amplified microsatellites at 6 loci (Table 2). In total, 90 alleles were observed, indicating a high degree of allelic diversity at these loci. Five of the six loci had null alleles with frequencies ranging from 0.0127 to 0.2342. Four loci showed significantly lower levels of observed heterozygosity than expected. The HWE test was conducted for each locus within the total population (Table 2) and within each population (S3 Table). All 6 loci deviated significantly from the HWE after Holm–Bonferroni sequential correction within the total population (Table 2) and within the Aichi, Shizuoka and Gifu populations (S3 Table). In the Oita population, we found deviation from the HWE in the EM-GGA1 and EM-AGGCA loci. The other populations revealed no significant deviation from the HWE (S3 Table). The pairwise linkage disequilibrium data calculated using Genepop revealed linkages between pairs of loci, but these linkages were not consistent across all geographic areas. The populations from Aichi, Shizuoka, and Gifu were found to have linkages between their loci, while other populations did not show any linkages (Table 3, S4 Table).

**Table 2:** Summary of genetic variation and fixation index from each selected microsatellite locus.

Locus	Microsatellite motif	Tm(°C)^a^	No.of alleles	No.of unique alleles	Ho^b^	He^c^	Fst^d^	HWE^e^	Null Alleles	GenBank accession number
EM-CTT1	(CTT)n	50	10	4	0.762	0.594	0.085	0.00*	-0.1697	MH978898
EM-AGC1	(AGC)n	60	17	1	0.718	0.805	0.055	0.00*	0.0367	MH978902
EM-GCAGAC	(GCAGAC)n	60	13	4	0.377	0.557	0.119	0.00*	0.1539	MH978899
EM-GGA1	(GGA)n	60	21	9	0.535	0.739	0.145	0.00*	0.1516	MH978900
EM-GGA2	(GGA)n	60	13	6	0.399	0.753	0.333	0.00*	0.2342	MH978901
EM-AGGCA	(AGGCA)n	60	16	3	0.800	0.802	0.064	0.00*	0.0127	MH978897

^a^:Tm(°C): Annealing Temperature

^b^: observed heterozygosity

^c^: Expected heterozygosity

^d^: Fixation index

^e^: Hardy Weinberg Equilibrium for total population

*: Significantly deviated from HWE after Holm-Bonferroni sequential correction

**Table 3:** Summary of linkage disequilibrium analysis.

	EM-AGC	EM-CTT1	EM-GCAGAC	EM-GGA1	EM-GGA2	EM-AGGCA
**EM-AGC**		A, S	A, S	A, S	A, G, S	A, S
**EM-CTT1**	+		A	A	A, G	A
**EM-GCAGAC**	+	+		A, G	A, S	A
**EM-GGA1**	+	+	-		A, G	A, G
**EM-GGA2**	+	+	+	-		A, G, S
**EM-AGGCA**	+	+	+	+	+	

The locus shown linkage after Holm–Bonferroni sequential correction was indicated by (+) the population which the linkage occur were A (Aichi), G (Gifu), and S (Shizuoka).

The analysis of molecular variance was done by assigning each isolate to a population based on geographic origin. The fixation index for each locus was between 0.055 and 0.333 (Table 4). These indicate low to moderate genetic differentiation between populations, suggesting that gene flow occurs between populations.

**Table 4:** Summary of analysis of molecular variance.

Source	df^a^	SS^b^	MS^c^	Est. Var.^d^	%^e^
Among Pops	15	184.574	12.305	0.435	19%
Within Pops	438	817.039	1.865	1.865	81%
Total	453	1001.612		2.300	100%
Among Cluster	2	102.334	51.167	0.322	14%
Within Cluster	461	926.070	2.009	2.009	86%
Total	463	1028.403		2.330	100%

^a^: degree of freedom

^b^: sum of square

^c^: mean of square

^d^: estimated variance

^e^: percent of variance

An index of association test revealed that some of the *P*. *helicoides* populations were highly clonal while the others were sexual (Table 5). The clonal populations are Aichi, Gifu, Oita, Shizuoka, and Yamanashi while the sexual popualtion include Iriomote, Kagawa, and Nara. Other populations could not perform the index of association test due to the low number of isolates.

**Table 5:** Genotypic diversity statistic of *Phytopythium helicoides* isolates used in this study.

Population	N	MLG^a^	e-MLG^b^	I_A_^e^	p.I_A_^d^	${\bar{r}}_{d}$^e^	p. ${\bar{r}}_{d}$^f^
Aichi	70	48	9.32	0.8303082	0.001	0.1689158	0.001
Fukui	2	1	1	NA	NA	NA	NA
Gifu	64	52	9.44	0.800526	0.001	0.1651008	0.001
Iriomote Island, Okinawa	8	8	8	0.1279318	0.301	0.0260981	0.303
Kagawa	4	4	4	0.8918919	0.189	0.3293151	0.076
Kushu lake, Rebun Island, Hokkaido	1	1	1	NA	NA	NA	NA
Mie	2	2	2	NA	NA	NA	NA
Miyagi	2	2	2	NA	NA	NA	NA
Nagano	1	1	1	NA	NA	NA	NA
Nara	4	4	4	0.2988506	0.253	0.0795255	0.259
Netherlands	1	1	1	NA	NA	NA	NA
Niigata	1	1	1	NA	NA	NA	NA
Oita	17	8	5.48	1.1565715	0.001	0.4390354	0.001
Saga	2	1	1	NA	NA	NA	NA
Shizuoka	34	31	9.59	0.5266307	0.001	0.1063102	0.001
Tochigi	3	3	3	-0.5	0.684	-0.3	0.965
Toyama	3	2	2	NA	NA	NA	NA
USA	2	2	2	NA	NA	NA	NA
Wakayama	2	1	1	NA	NA	NA	NA
Yamagata	1	1	1	NA	NA	NA	NA
Yamanashi	8	6	6	1.4624374	0.002	0.2961952	0.001

a. Multi Locus Genotypes

b. estimated Multi Locus Genotype

c. Index of association

d. P value for Index of association (I_A_)

e. alternative value of index of association (${\bar{r}}_{d}$)

f. P value for Index of association (${\bar{r}}_{d}$)

### Population structure analysis

**Fig 2:**
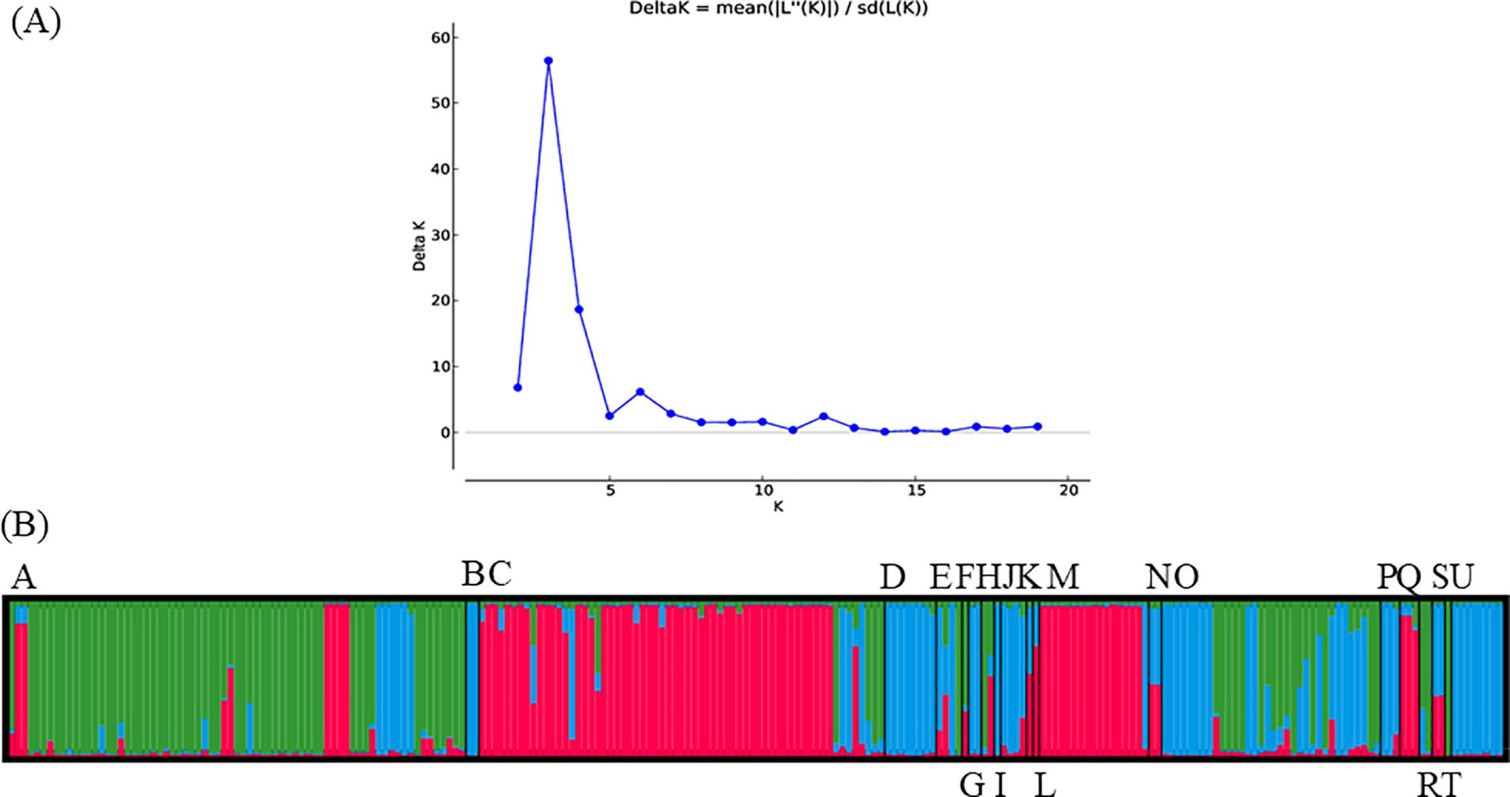
STRUCTURE analysis of 232 isolates of *P*. *helicoides*. **(A) ΔK was calculated using STRUCTURE HARVESTER, and the results indicated that the most likely number of genetic clusters (K) was 3. (B) Histogram showing the estimated proportions of genetic clusters in each of 21 populations, based on K = 3 clusters (red, green, and blue).** A: Aichi; B: Fukui; C: Gifu; D: Okinawa; E: Kagawa; F: Hokkaido; G: Mie; H: Miyagi; I: Nagano; J: Nara; K: Netherland; L: Niigata; M: Oita; N: Saga; O: Shizuoka; P: Tochigi; Q: Toyama; R: USA; S: Wakayama: T: Yamagata; U: Yamanashi.

The population structure analysis was performed using the STRUCTURE software. STRUCTURE uses a model-based clustering method that can accurately cluster individuals into genetic groups by estimating different numbers of clusters (K). The STRUCTURE HARVESTER then processes the STRUCTURE results to determine the best K that fits with the data. CLUMPP is used to align the cluster assignments across replicate analyses and the results are then visualized by DISTRUCT[21]. The clustering analysis performed suggested that K = 3 is the most likely scenario for all samples tested (Fig 2A, S5 Table). The K = 3 scenario was applied to all 21 geographic populations. The results highlighted the divergence between the Gifu, Oita, and Toyama populations (mainly red) and the other populations (mainly green and/or blue) (Fig 2B). The Aichi population consisted of all 3 genetic groups with green as the majority. The Shizuoka population had two genetics groups (green and blue) with blue as the majority. The Tochigi, Yamanashi, and Iriomote populations also clustered in the blue genetic group while the Kagawa, USA, and Yamagata populations belonged mainly to the red genetic group (Fig 2B). Pie diagrams showing the distribution of each genetic cluster within the 19 prefectures in Japan also revealed the dominancy of a particular group in most prefectures (Fig 1). Miyagi, Yamagata, Rebun Island, and Aichi were dominated by the green group; Gifu, Toyama, Niigata, and Oita were dominated by the red group; and other regions were dominated by the blue group except for Shizuoka, which had similar amounts of blue and green.

**Fig 3:**
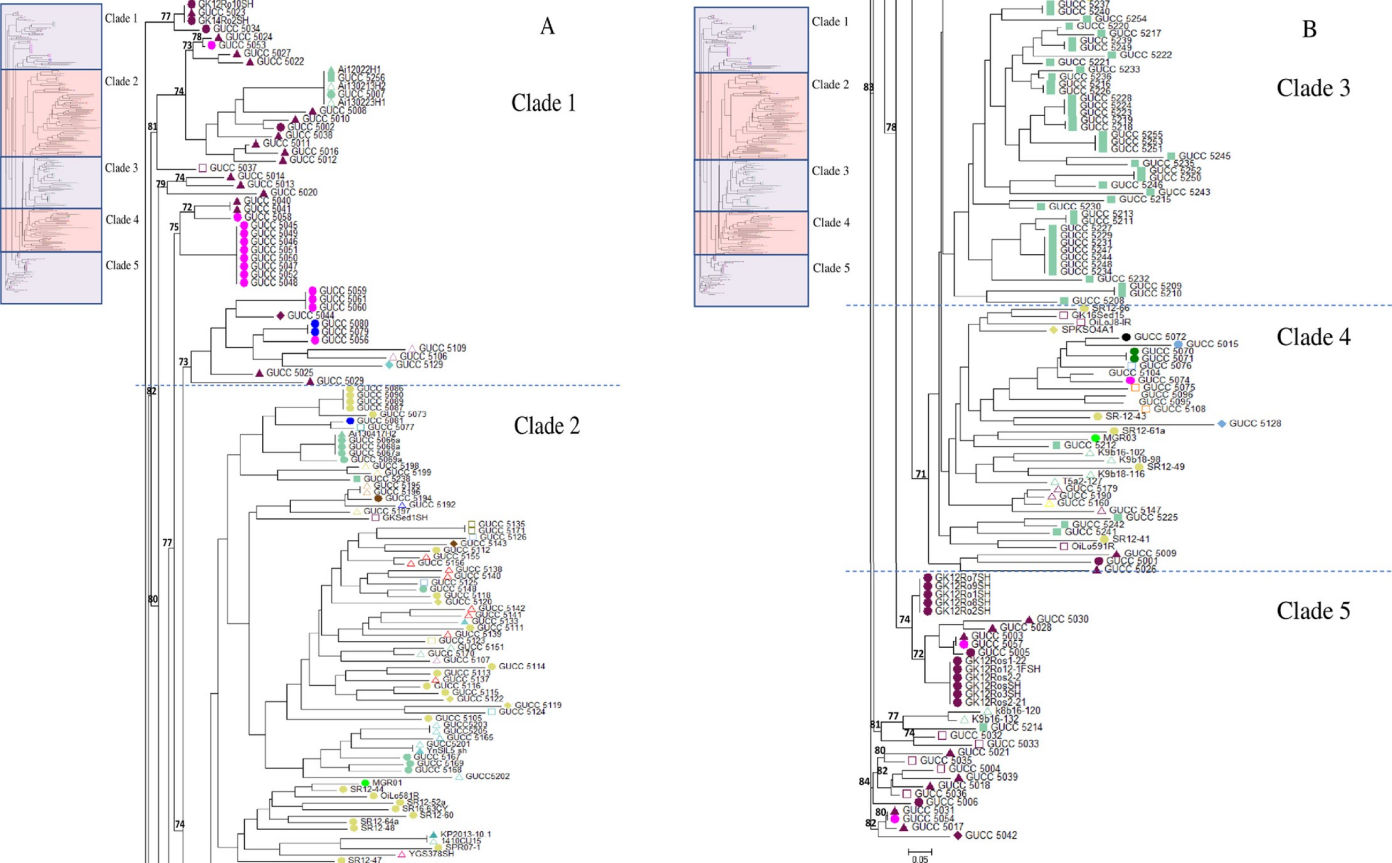
**A phylogenetic tree of the *P*. *helicoides* populations in Japan (A) clade 1–2; (B) clade 3–5.** The phylogenetic tree was constructed using neighbor joining algorithm. The mode labels show percentage of probability bootstrap probability in 1000 replications. Hosts: ■ poinsettia; ▲ miniature rose; ● rose; ♦ strawberry; Δ natural environment; □ others. Geographic origin (by prefecture): ■ Aichi; ■ Fukui; ■ Gifu; □ Hokkaido ■ Iriomote; ■Kagawa; ■ Mie; ■ Miyagi; ■ Nagano; ■ Nara; ■ Niigata ■Oita; ■ Saga; ■Shizuoka; ■ Tochigi; ■ Toyama; ■ Wakayama ■ Yamagata ■ Yamanashi.

The clustering results were supported by the phylogenetic tree, which divided all the samples into 5clades (Fig 3). The individuals belonging to the green cluster in STRUCTURE were mainly allocated to clades 2 and 3 on the tree; individuals from the red cluster were in clades 1 and 5, while those from the blue cluster were mainly in clade 4. On the tree, the prefecture origin of each isolate is indicated by color (Fig 3) The first and fifth clade was majority contained with isolates collected from Gifu and Oita. The third clade consisted only of isolates from poinsettia in Aichi. The second and fourth clade consisted of isolates collected from Shizuoka and other populations.

Host origin is indicated by shape on the phylogenetic tree (Fig 3). Isolates from rose and miniature rose tended to group together. In the first and fifth clade of the tree, isolates from cutting rose in Oita grouped with isolates from rose and miniature rose in Gifu. The isolates from miniature rose in Aichi shared the same branch with an isolate from miniature rose in Gifu. In the second clade, isolates from rose in Shizuoka grouped with isolates from rose in Aichi. Isolate 188 from Shizuoka and all the isolates from Aichi were asexual.

The isolates from natural environments were also scattered among clades. In some cases, they were grouped with isolates from agricultural areas in the same geographic regions. For example, in clade 1 the isolates from Aichi water were closely related to an isolate from rose around Aichi. In other cases, the isolates from natural environments grouped more closely with isolates from natural environments in different geographic regions. The isolates collected from soil in Gifu were grouped on to the fourth clade while most of the isolates collected from the infected plant around the area were in the first and fifth clade.

## Discussion

Knowledge about the genetic diversity of *P*. *helicoides* has been very limited. Our previous study uncovered intra-isolate variation in the rDNA ITS region [12]. Microsatellite markers have been used to characterize the population structures of *Phytophthora cinnamomi* [21], *Ph*. *infestans* [30], *Ph*. *sojae* [31], *Ph*. *nicotianae* [32], and *Ph*. *ramorum* [33]. The use of microsatellite markers for *P*. *helicoides* was initiated by Yin-Ling et al (2009), but in that study only 3 isolates were used to check the reliability of the primers.

In this study we used suppression PCR and TAIL-PCR to develop 7 novel microsatellite markers, and 6 of these were suitable for population genetics analyses (Table 2). In total, 90 alleles were obtained from the 6 markers, indicating that the loci were highly polymorphic and suitable for population genetics studies. Some of the loci were found out to have null alleles. However, the number is relatively low (0.0127 to 0.2342). Null alleles with frequencies in this range will not affect individual assignments in clustering analyses [23]. As a homothallic species, *P*. *helicoides* is generally assumed to perform sexual reproduction by self-fertilization so it was expected to deviate from HWE. This phenomenon is common in oomycetes such as *Pythium ultimum* [34], *Ph*. *plurivora* [35], and *Pseudoperonospora cubensis* [36]. The LD test revealed a significant deviation from linkage disequilibrium on the some loci found in Aichi, Gifu, Oita, and Shizuoka populations. However, the linkage were not consistent across all populations. This result was related to the index of association test result (Table 5) that suggested the population of *P*. *helicoides* from Aichi, Gifu, Oita, Shizuoka and Yamanashi were highly clonal. These isolates were collected mainly from rose and miniature rose and also included several asexual isolates (S1 Table). The index of association is critical to determine the clonality of a population. It is obtained in R package poppr by resampling of the data to obtain a null distribution for the expectation of random mating [37]. The software calculated the index of association (I_A_) and the standardized index of association (${\bar{r}}_{d}$) for comparison [20]. The standardized index of association was formulated to resolve the issue of scaling with increasing number of loci [38]. The presence of repeated genotypes will increase the associations among loci which is one of the most obvious signatures of clonal reproduction [39]. From these calculations, the populations suggested to have sexual reproduction based on the index of association are Kagawa, Nara, and Iriomote, the isolates were collected from chrysanthemum, soil, and water, respectively. The highly linkage microsatellite loci due to clonal reproduction also found in *Ph*. *cinnamomi* [21], *Ph*. *ramorum* [40] [41], and three species formerly *Ph*. *citricola* complex, *Ph*. *plurivora*, *Ph*. *multivora*, and *Ph*. *pini* [42]. The clonality on the *P*. *helicoides* populations that found on the isolates causing root rot suggested that the actively infecting pathogen could spread clonally by asexual structure, zoospores, while it could produce sexual structure, thick walled oospore, to make recombination and to survive for a long time after host plant death. Furthermore, the isolates collected from natural environment will serve as genetic reservoir [43]. The fixation index (Fst) data (Table 2) also suggested the possibility of gene flow between populations, indicating that migration and outcrossing could play important roles in the development of genetic diversity among individuals of *P*. *helicoides*. This result is supported by the study on rDNA ITS diversity in *P*. *helicoides* [12]. Outcrossing of homothallic species is also observed in *Py*. *ultimum*[34], *Py*. *irregulare* [44], and *Phytophthora sojae* [45].

The clustering analysis performed using STRUCTURE and the phylogenetic analysis showed clear congruence; even though the STRUCTURE analysis found three genetic groups while the tree contained 5 clades. The first and fifth clades contained many isolates from rose and miniature rose in Gifu, Oita, and Aichi; these isolates were mainly found in the red cluster in STRUCTURE. Isolates in the green cluster were allocated to the second clade, which contained several Aichi and Shizuoka isolates, and the third, which consisted exclusively of isolates from poinsettia in Aichi. The fourth clade consisted mainly of natural environmental isolates and isolates from other hosts that are not the major focus in this study; these were mainly in the blue cluster in STRUCTURE.Studies of *Ph*. *infestans* in China [46] and *Ph*. *austrocedrae* in Argentina [47] also found clustering based on geographical origin. In the STRUCTURE analysis, we assumed admixture in populations that had less than 70% of one genetic group [46], suggesting that the Wakayama, Saga, and Nagano populations are admixed. These were formed by gene flow between two or more genetically distinct populations [48].

Several *P*. *helicoides* isolates used in this study are asexual and therefore classified as Group P[13]. These isolates have the ability to produce abundant quantities of zoospores. In the STRUCTURE analysis, asexual isolates with the same geographical origin were allocated to the same genetic groups. This was supported by our previous study, in which Group P isolates showed different banding patterns than those of a sexual strain in an RFLP analysis of the rDNA ITS region [12].

*Phytopythium helicoides* from natural environments could be an important source of disease outbreaks in nearby farms. On the phylogenetic tree, several isolates from natural environments were grouped with isolates from nearby agricultural areas. The isolates from irrigation water in Aichi were grouped with other Aichi isolates in both the STRUCTURE and phylogenetic analyses. Interestingly, a *P*. *helicoides* outbreak occurred on a farm downstream of a putative natural source in Aichi, but not on a closer farm. This suggested that the *P*. *helicoides* strain could have been carried to the farms by river. Other isolates from irrigation water in Aichi were closely related to isolates from Gifu. There are rivers that flow from Gifu to Aichi, so it is possible that this water-borne disease was carried to Aichi by river. Our data suggest that *P*. *helicoides* might be native to many areas, even though it has only recently caused disease outbreaks. This may be due to the increasing use of hydroponics culture, which favors the spread of *P*. *helicoides*. Another factor might be global warming, which would encourage the growth of this high temperature tolerant species. The phylogenetic tree also supported the result obtained from STRUCTURE as the isolates collected from farms were show close relationship with the isolates collected from the natural environment in the same geographic areas.

This study indicated that the host plants could have a significant influence on the population genetics of *P*. *helicoides*. The isolates from cutting rose in Gifu and Oita showed closer relationships than isolates from miniature rose in Gifu (Fig 3). This may be due to differences in the cultivation systems of cutting rose and miniature rose. Cutting roses are grown in hydroponic culture systems, while miniature roses are grown in potting media. It is possible that the pathogen is transported between farms in the hydroponic nutrients for cutting rose or the potting mix for miniature rose.

In conclusion, we found that *P*. *helicoides* has high variance within individuals, indicating a high degree of heterozygosity and the ability for outcrossing. The 6 loci tested in this study showed low fixation indices and deviated significantly from the HWE, suggesting the occurrence of gene flow between populations. The isolates collected from farming area are highly clonal while the isolates from natural environment indicated the occurrence of sexual reproduction. The migration of the pathogen could be facilitated naturally, in drainage systems, or by human activity in the transportation of agricultural materials.

## Supporting information

S1 Table Isolates of *Pythopythium helicoides* used in this study. (XLSX)

S2 Table TAIL-PCR primers used in this study. (DOCX)

S3 Table Genetic variation among each locus for each population. (XLSX)

S4 Table Results from GenePop. (XLSX)

S5 Table Output from Structure Harvester. (XLSX)
