# Supplementary material for: Population structures of the water-borne plant pathogen Phytopythium helicoides reveal its possible origins and transmission modes in Japan
Source: PLoS One. 2018 Dec 26;13(12):e0209667. doi: 10.1371/journal.pone.0209667 (PMC6306214; doi:10.1371/journal.pone.0209667)
Supplement: S2 Table — (DOCX) [file pone.0209667.s002.docx]

**S2 Table. TAIL-PCR primers used in this study**

| **Microsatellite Motif** | **Primer Name** | **Primer sequence (5'-3')** |
| --- | --- | --- |
| (AGC)n | H5AGC1a | CGATGGCTGCACATAGCGTTGTTC |
|  | H5AGC1b | TGCAACTGCGATGGCTGCACATAG |
|  | H5AGC1c | TGTGGGCTCTCGCGATTTGTC |
|  |  |  |
|  | H5AGC2a | TCTAGCACGCTGGTTACCGCCTC |
|  | H5AGC2b | ACCTTGGCGACGACTTTGCTG |
|  | H5AGC2c | GTACCGTAAGTGCATTCCGCG |
|  |  |  |
|  | H5AGC3a | TGTCATCCCACCACGACACCTC |
|  | H5AGC3b | CCTTCGAAACCCTGTCATCCCAC |
|  | H5AGC3c | CCACCTTCGCGCAACATCTTC |
|  |  |  |
|  | H5AGC4a | TGCGTCTGCATCTGTGCGTG |
|  | H5AGC4b | TGTTGGCGTGCGTCTGCATC |
|  | H5AGC4c | GCTGCTGACCGACACGTA |
|  |  |  |
|  | H5AGC5a | TTCGCCGGTGACTTTCCGTG |
|  | H5AGC5b | ATGGAGGCTTCTCCGCCATCTTC |
|  | H5AGC5c | CTTGGGATGGAGGCTTCTCC |
|  |  |  |
|  | H5AGC6a | CCATCATGGGATAACGTCCCGAG |
|  | H5AGC6b | CCTGAAGTGCTCAATCTTCAACCGG |
|  | H5AGC6c | GCAACAATGCGCCACCGAAC |
|  |  |  |
|  | H5AGC7a | GCGGAAACGCAGACTCGATC |
|  | H5AGC7b | CTCCATCAACATCTCCACGCGC |
|  | H5AGC7c | CGACCTCGTGGCTCTTCCATAC |
|  |  |  |
|  | H5AGC8a | TTGGTGACGTGCTCTGCCTC |
|  | H5AGC8b | CGTCGCTGTGCTCAACCTTG |
|  | H5AGC8c | AAGGCCCTGCTTCGATTGAGGAC |
|  |  |  |
|  | H5AGC9a | ATTAGCCTCCACGCGCTGCT |
|  | H5AGC9b | TTTTGGCGCAGGGAAACGCCTC |
|  | H5AGC9c | TCGGCTGCATGCCCGTTTTG |
|  |  |  |
| (CAA)n | H5CAA1a | TGGGCGACCGCTTCTTTTG |
|  | H5CAA1b | TCAGTCGGTAGAGCGCAAGAC |
|  | H5CAA1c | GACTCTTAATCTTGTGGTCGTGGG |
|  |  |  |
|  | H5CAA2a | GCCGACCATCGGTGTGTAGATAATC |
|  | H5CAA2b | ATGCCAACGACAAGGCACAAGGCG |
|  | H5CAA2c | GGTGCGTCTTGGGGTTGAAAAC |
|  |  |  |
| (TCA)n | H5TCA1a | CGAAGACGTGGAACTTGTCCG |
|  | H5TCA1b | ACAACGCCGAGGGTCGAATC |
|  | H5TCA1c | AACATCACTGGCCAGCAGAGC |
|  |  |  |
|  | H5TCA2a | GATCAGCACTGGAACACCACTTC |
|  | H5TCA2b | GTCATGACTTCAGATCAGCACTGG |
|  | H5TCA2c | AGGGCTCGACGAAGATGATGAC |
|  |  |  |
|  | H5TCA3a | TAAGGAGGCTGCTGTGCGTG |
|  | H5TCA3b | GGAGACTTGTCGCTGTGCTTG |
|  | H5TCA3c | TGTTGTTGCCATCGCCATCG |
|  |  |  |
|  | H5TCA4a | TGTGCAAAGCATCGACAGCAG |
|  | H5TCA4b | CACCATCACTCCCAAAGTCAGG |
|  | H5TCA4c | GACCAATCTTCACTCTTTGGGGC |
|  |  |  |
|  | H5TCA6a | TTGCGGCTCAAACACTCTGC |
|  | H5TCA6b | AAAACGCCGTCTGAGGCTTG |
|  | H5TCA6c | CATTCCAAGTCAATCTGCCACCG |
|  |  |  |
|  | H5TCA7a | GGCACAAAGCACGACACACTTG |
|  | H5TCA7b | CCGTAGCACACGGCACAAAG |
|  | H5TCA7c | TACGTTGGCTTACTCGCCCG |
|  |  |  |
| (CAA)n | H5CAA3a | GTAGCCCACTCATTGAACCCAAGC |
|  | H5CAA3b | TAACACGCCAACACGATGCCTG |
|  | H5CAA3c | TTGACGAATGGGAGCCTCCACTAC |
|  |  |  |
|  | H5CAA4a | GCGTTGATGCGTTGATGTGATG |
|  | H5CAA4b | GCTTGTTTGGTGTCGGGTGTATGTG |
|  | H5CAA4c | GCTTGTGTGGTGTGTGAGGAAGTG |
|  |  |  |
| (CTT)n | H5CTT1a | GAAAAGACGATGAGGACGATGGTG |
|  | H5CTT1b | ACGATTCCGGTTCGACTTGC |
|  | H5CTT1c | GCAAAGAAGACGAACGGGACG |
|  |  |  |
|  | H5CTT2a | ATGGACTGCAGGAGGAGAAG |
|  | H5CTT2b | TTCGTGCGCCAACGAACCAG |
|  | H5CTT2c | TGTGCAGGTGGTGCTTCGTG |
|  |  |  |
|  | H5CTT3a | CCCAGTGCTTCAAACGAAGAGG |
|  | H5CTT3b | GAAGAGAGTCTGCACCCAGTGC |
|  | H5CTT3c | CGAGTCTCGGAAAGTGCTGCTTC |
|  |  |  |
|  | H5CTT4a | ATGGGGCAAGTCCAGCCCAAAAG |
|  | H5CTT4b | TTGATGGGGCAAGTCCAGCC |
|  | H5CTT4c | GCGACGACGATTTTGATGGGGC |
|  |  |  |
|  | H5CTT5a | GCGACAACATGGATGCTCGTG |
|  | H5CTT5b | AGTTGATGGCGAGAGCGACAAC |
|  | H5CTT5c | TTGTGGGTTCCTCCGTGGTTC |
|  |  |  |
|  | H5CTT6a | AGCGAGTCAAAGAGCGCAGTG |
|  | H5CTT6b | ACCAGTGCAGTAGGTTGCGAG |
|  | H5CTT6c | ATCAGGATGCGACGCTCACAG |
|  |  |  |
|  | H5CTT7a | CACACTACCGAAACGACGCCATC |
|  | H5CTT7b | CACACGCACAAAAACACACTACCG |
|  | H5CTT7c | CGAACATACTCACACACACGCAC |
|  |  |  |
| (GGA)n | H5GGA1a | TGCTGTCGATACGTGCTGCTC |
|  | H5GGA1b | TTTCGTCGTCCTCGTGCTCTTC |
|  | H5GGA1c | CCTCGTCGAAGGCGTCACTTTC |
|  |  |  |
|  | H5GGA2a | AAAGGTGGGAGCACCACCTGTG |
|  | H5GGA2b | CTTCTTGGAGGAAAGGTGGGAGC |
|  | H5GGA2c | CCCCAACTCTGGGTCGTTAATC |
|  | H5GGA3a | AGGAAGAGGAAGGCCTGCTG |
|  | H5GGA3b | ATCGCTGCGTGTCCATTCCGAG |
|  | H5GGA3c | ATCGCTGCGTGTCCATTCCGAG |
|  |  |  |
|  | H5GGA4a | TGTGCCCACGACCTCAAAGC |
|  | H5GGA4b | TCGCTTTAGCGACGAGGGAG |
|  | H5GGA4c | TCGCTTTAGCGACGAGGGAG |
|  |  |  |
|  | H5GGA5a | ACGATCCCTCCGCCATATCC |
|  | H5GGA5b | ATCTCGGCTACCTGTGCCTC |
|  | H5GGA5c | ACCACCGTTGGACCCATCTC |
|  |  |  |
|  | H5GGA6a | CTTCGTCATCGTCGTCTCCCTC |
|  | H5GGA6b | TCCTCGTCGTCCTCTTCGTCTTC |
|  | H5GGA6c | TCATCGTCGTCCTCCTCGTC |
|  |  |  |
|  | H5GGA7a | ACATCCTCTCCACCTCCGCTTG |
|  | H5GGA7b | TGCTGCCTTGACACCTCCAG |
|  | H5GGA7c | CCAGCTTCTCCACCTTCACCAC |
|  |  |  |
|  | H5GGA8a | GGAAGCACCACAGTCACCAC |
|  | H5GGA8b | ACTAGCAGCACCGGAAGCAC |
|  | H5GGA8c | CCGATCTTTGCGGCCACTAG |
|  |  |  |
|  | H5GGA9a | GAACGGGTTGTCGTCCACTTC |
|  | H5GGA9b | AAACTCGCGCACATCCTCCGTC |
|  | H5GGA9c | TCGGTCCTTGGGTTTCACGG |
|  |  |  |
|  | H5GGA10a | AAAGCGGCACTACCACAGCC |
|  | H5GGA10b | GCTGCTTCTTCGACTCAAAGCG |
|  | H5GGA10c | CGGCTGTCGAGTTTCTGACC |
|  |  |  |
|  | H5GGA11a | TTTCGGCTTCACCGCCTGCTTC |
|  | H5GGA11b | CGAGCGACTCGACACCTCTTTC |
|  | H5GGA11c | TTTCTCGCCTCGTCACCTCC |
|  |  |  |
|  | H5GGA12a | GCGACCTCGTGAAACTCTACCTC |
|  | H5GGA12b | AGCGCGACCTCGTGAAACTC |
|  | H5GGA12c | AGCGCGACCTCGTGAAACTC |
|  |  |  |
|  | H5GGA13a | TGTGCGATGTTGTCGCCCAG |
|  | H5GGA13b | GTTCACCACCATCAGCCGAC |
|  | H5GGA13c | GTCCCTGCCAAGTTCACCAC |
|  |  |  |
|  | H5GGA14a | TTACCATGGCGACGACTCCG |
|  | H5GGA14b | TGGTGGATGGATCTCTTCAACCTAC |
|  | H5GGA14c | GCCCTGGTGGATGGATCTCTTC |
|  |  |  |
|  | H5GGA15a | CTTTTGCCGCGGCTTCTTGG |
|  | H5GGA15b | TTGTTGATCGGGCTGTTCCCAG |
|  | H5GGA15c | GTCATGGAGGCTTGTTGATCGG |
|  |  |  |
